# Reduced DTI-ALPS in H-type hypertension: insights into perivascular space function

**DOI:** 10.3389/fneur.2025.1536001

**Published:** 2025-04-04

**Authors:** Chun Zhang, Caihong Song, Shiying Sheng, Liang Pan, Lingling Sun, Wei Xing

**Affiliations:** ^1^Department of Radiology, Third Affiliated Hospital of Soochow University, Changzhou, China; ^2^Department of Radiology, Huai’an Maternity and Child Healthcare Hospital, Huai’an, China; ^3^Department of Laboratory Medicine, The Fifth People's Hospital of Huaian, Huai’an, China; ^4^Department of Neurology, Third Affiliated Hospital of Soochow University, Changzhou, China

**Keywords:** hypertension, homocysteine, glymphatic function, diffusion imaging, vascular cognitive impairment

## Abstract

Recent studies suggest that glymphatic dysfunction plays a significant role in vascular cognitive impairment (VCI). Both hypertension and hyperhomocysteinemia are independent risk factors for VCI, and their combination is referred to as H-type hypertension (HHT). However, the impact of HHT on glymphatic function remains unclear. This study used the recently popular indirect marker, diffusion tensor imaging along the perivascular space (DTI-ALPS) to assess potential changes in glymphatic function in patients with HHT. We recruited 58 HHT patients and 50 healthy controls without hypertension, collecting clinical, cognitive, biochemical, and diffusion MRI data. Behaviorally, HHT patients scored lower on global cognitive tests compared to controls. DTI-ALPS analysis revealed a bilateral reduction in DTI-ALPS in HHT patients. Correlation analysis showed strong associations between lower DTI-ALPS values, reduced cognitive scores, and elevated homocysteine (Hcy) levels in HHT patients. Mediation analysis further indicated that DTI-ALPS largely mediates the relationship between Hcy levels and cognitive performance. These findings suggest that hypertension and elevated Hcy levels contribute to DTI-ALPS reduction, which may underlie the cognitive decline observed in HHT.

## Introduction

Hypertension is a clinical condition affecting over one-third of the global adult population (1). According to the American College of Cardiology guidelines, hypertension is defined by a systolic blood pressure ≥ 130 mm Hg, a diastolic blood pressure ≥ 80 mm Hg, or the use of antihypertensive medication (2). It is a leading risk factor for cardiovascular diseases and premature death. In China, approximately 75–80% of cases of essential hypertension are classified as H-type hypertension (HHT), a prevalence significantly higher than in Western countries. HHT is characterized by the combination of hypertension and elevated homocysteine levels (Hcy ≥ 10 μmol/L) (3). Compared to hypertension alone, HHT is associated with a 25-30-fold increased risk of cardiovascular events and a 12-fold increased risk of stroke (4). These factors make HHT a distinct risk factor for vascular cognitive impairment (VCI), yet the neural mechanisms underlying cognitive decline in HHT remain poorly understood.

HHT negatively impacts brain vasculature, parenchyma, and metabolism, increasing the risk of cognitive decline. Neuroimaging has provided valuable insights into how HHT affects brain structure and function. Previous studies have shown that elevated homocysteine levels are linked to increased amyloid-beta (Aβ) plaques and neurofibrillary tangles, cortical thinning (5), higher white matter hyperintensities (6, 7), and reduced white matter volume (8, 9). These changes contribute to cerebral small-vessel disease by damaging endothelial cells (10, 11), accelerate brain aging (12), and serve as independent risk factors for cognitive impairment in older adults (13). Additionally, studies have suggested that hypertension is associated with cortical hypoperfusion (14), widespread gray matter loss (5, 15), changes in default network morphology (16) and functional connectivity (17), as well as disruption of the frontoparietal network (18) and white matter integrity (19). Together, these findings suggest that both hypertension and elevated homocysteine levels have similarly detrimental effects on brain structure and function. However, the extent to which HHT impacts brain function and the neural mechanisms underlying cognitive impairment remain poorly understood.

The glymphatic system is a brain-wide metabolic waste clearance system (20). It facilitates the exchange of cerebrospinal fluid (CSF) with interstitial fluid (which surrounds brain cells) to clear metabolic waste via perivascular spaces surrounding blood vessels, particularly *tau* proteins and amyloid-like proteins, playing a critical role in the pathogenesis of neurodegenerative diseases, such as Alzheimer’s disease (21). Previous studies have indicated that stroke and other forms of brain injury can impair the glymphatic system, leading to dysfunction in waste clearance (22, 23). As a marker of brain injury, the glymphatic system has the potential to identify damage in conditions like HHT and may correlate with cognitive decline. Its role in protein clearance and fluid exchange suggests it could be a key factor in understanding brain pathology and VCI (20, 24).

Diffusion tensor imaging along the perivascular space (DTI-ALPS) offers a noninvasive method for studying fluid movement in the perivascular space, potentially reflecting aspects of the glymphatic system (25, 26). This technique has demonstrated strong stability and consistency in assessing fluid movement in the perivascular space (27, 28), making it a viable alternative to invasive procedures in clinical practice. A recent study has shown that DTI-ALPS primarily reflects fluid drainage, as evidenced by contrast enhancement (29). It has been used to identify impairments in conditions such as sleep disorders, traumatic brain injury, Alzheimer’s disease, and stroke, often correlating with cognitive performance (22, 25, 28, 30, 31).

In the context of HHT, ALPS provides an excellent opportunity to assess glymphatic dysfunction in this patient population, especially when compared to primary hypertension alone. This study aims to explore whether HHT is associated with reduced ALPS values and to investigate the relationship between ALPS, Hcy levels, and cognitive performance. We hypothesize that HHT will show more significant reductions in ALPS, which will be linked to cognitive impairment in these patients.

## Materials and methods

### Participants

The HHT patients recruited for this study were individuals identified during hospital health check-ups, with most being first-time cases of this condition. Consequently, they were promptly enrolled in our study. After completing data collection, the majority of these patients have since received conventional medical treatment or made proactive lifestyle adjustments. Sixty-two HHT patients and 51 normotensive healthy controls were enrolled in this study. Informed consent was obtained from all participants, and the study was approved by the Ethical Committee for Human Research at Changzhou First People’s Hospital, in accordance with the Declaration of Helsinki.

A physician (author CZ) performed the diagnosis and examination for all participants. Each participant underwent a comprehensive neurological evaluation, as well as blood tests and neuropsychological assessments. The inclusion criteria for HHT patients were: (1) age between 18 and 65 years; (2) right-handedness; (3) diagnosis of HHT, defined by serum Hcy levels >10 μmol/L and clinical systolic blood pressure (SBP) > 140 mmHg and/or diastolic blood pressure (DBP) > 90 mmHg; (4) no current antihypertensive treatment; and (5) no contraindications for MRI.

Exclusion criteria for all participants included: (1) any history of cardiovascular or cerebrovascular events; (2) significant prior cerebral infarction or hemorrhage; (3) major health conditions, including psychiatric disorders, renal failure (serum creatinine >176 μmol/L), liver damage (aspartate aminotransferase (AST) or alanine aminotransferase (ALT) > 40 IU/L), coronary heart disease, congestive heart failure, or a history of malignant tumors; (4) other structural abnormalities that could affect cognitive function; and (5) education level lower than 6 years.

### Demographic, clinical, and cognitive data acquisition

Hypertension was defined as seated, resting SBP ≥ 140 mmHg and/or DBP ≥ 90 mmHg, or a self-reported history of hypertension. Elevated Hcy levels were defined as ≥10 μmol/L.

Blood pressure measurements were taken by two trained researchers (authors) after each participant had rested in a seated position for at least five minutes. In addition to blood pressure, demographic and medical history information was collected (Table 1). Neurocognitive function was assessed using the Mini-Mental State Examination (MMSE) (45) and the Montreal Cognitive Assessment (MoCA) (32), which are widely used tests for evaluating global cognition.

**Table 1:** Demographics.

Demographic data	HHT (*n* = 58)	HC (*n* = 50)	Statistic, t/χ^2^ (*p*)
Age, mean (SD), y	48.96 ± 6.69	47.34 ± 6.90	1.238 (0.219)
Sex: males/females	32/26	25/25	0.288 (0.591)#
Education, mean (SD), y	11.304 ± 2.558	11.780 ± 2.957	−0.882 (0.380)
Smoking: *n* (%)	27/31	19/31	0.803 (0.370)^#^
SBP, mean (SD), mmHg	153.241 ± 7.828	118.100 ± 11.427	18.348 (<0.001***)
DBP, mean (SD), mmHg	99.379 ± 10.574	74.721 ± 7.570	14.065 (<0.001***)
Hcy (μmol/L)	24.073 ± 11.698	10.340 ± 2.858	8.646 (<0.001***)
LDL	2.775 ± 0.653	2.732 ± 0.669	0.335 (0.739)
HDLC	1.080 ± 0.282	1.220 ± 0.289	−2.491 (0.014)
Triglyceride	2.120 ± 1.012	1.647 ± 1.341	2.016 (0.047)
Total cholesterol	4.826 ± 0.924	4.897 ± 0.983	−0.378 (0.706)
Fasting blood glucose	5.771 ± 1.587	5.233 ± 1.304	1.904 (0.060)
HbA1c	6.443 ± 1.748	6.074 ± 0.944	1.195 (0.236)
Creatinine	72.168 ± 16.162	64.828 ± 13.060	2.572 (0.012)
Urea nitrogen	19.408 ± 106.899	5.1292 ± 1.200	0.999 (0.322)
CRP	6.9107 ± 8.121	6.4836 ± 10.025	0.169 (0.866)
MMSE	29.018 ± 1.168	29.740 ± 0.527	−4.176 (<0.001***)
MoCA	27.858 ± 1.354	28.900 ± 0.974	−4.585 (<0.001***)

Table shows mean (standard deviations) for all variables for hypertensive subjects and normotensive controls in this study. LDL, low-density lipoprotein; HbA1c, glycosylated hemoglobin, Type A1C; SBP, systolic blood pressure; DBP, diastolic blood pressure; ^#^represents the chi-square test. *p < 0.05; **p < 0.01; ***p < 0.001.

### MRI acquisition

MRI data were acquired on a 3.0 T MRI scanner (MAGNETOM Verio; Siemens Healthineers, Erlangen, Germany) at Changzhou First People’s Hospital, Jiangsu, China. The following sequences were used in this study: (1) whole-brain T1-weighted anatomical imaging (T1-MPRAGE): 3D sagittal acquisition, slice thickness = 1 mm, repetition time (TR) = 1900 ms, echo time (TE) = 2.52 ms, inversion time = 900 ms, flip angle = 9°, matrix size = 256 × 256; (2) diffusion tensor imaging (DTI): 2D acquisition, slice thickness = 3 mm, slice spacing = 1.875 mm, TR/TE = 3.8/0.102 s, flip angle = 90°, matrix size = 128 × 128, 64 directions with b-value of 1,000 s/m^2^ and one b = 0 s/m^2^, using GRAPPA-accelerated SS-EPI sequence and monopolar diffusion preparation. Routine MRI sequences, including T2-3D and susceptibility-weighted imaging (SWI), were also performed to rule out significant lesions, congenital abnormalities, or other conditions. The specific parameters for these routine clinical MRIs are not detailed here.

### DTI data processing

Each subject’s T1 and DTI images were first visually inspected by radiologists to ensure the absence of obvious abnormalities or lesions. After visual inspection, raw DTI data were entered into the preprocessing stream using the FMRIB Software Library (FSL Ver 6.4).^1^ Briefly, DTI data were corrected for susceptibility geometric, eddy current, and motion distortions. With both AP and PA coding images, TOPUP corrections were also administered. Diffusion tensor maps were then generated for the directions of the x- (right–left, D*_xx_*), y- (anterior–posterior, D*_yy_*), and z-axes (inferior–superior, D*_zz_*).

### Region of interest (ROI) placement

To improve the identification of individual ROI, we used the color fractional anisotropy map to define ROIs in each individual space. Specifically, we drew a 5 mm-radial spherical ROI on both sides of the projection fiber and the association fiber on the lateral ventricle. In the projection fibers, the dominant ones move in the z-axis (D*_zz_*), perpendicular to both the x- (D*_xx_*) and y-axes (D*_yy_*); while in the association fibers, the dominant ones run in the y-axis (D*_yy_*) direction, perpendicular to both the x- (D*_xx_*) and z-axes (D*_zz_*). Applying the spherical ROI, we extracted the average diffusion value inside the sphere to calculate the DTI-ALPS.

To ensure our results were not influenced by ROI selection, we also used an alternative, widely adopted atlas to calculate ALPS. As a supplementary validation, we compared the values generated by these two analyses and assessed the intraclass correlation coefficient (ICC).

### Statistical analysis

All descriptive and statistical analyses were performed using SPSS software (version 22, IBM Corp., Armonk, NY, USA). The *Chi*-square test was used to assess categorical variables (e.g., gender, diabetes, obesity, smoking, drinking), while continuous variables were analyzed using independent two-sample *t*-tests. A significance level of *p* < 0.05 was set for all tests.

### Mediation analysis

A mediation model was used to explore the complex relationships between ALPS, cognitive outcomes, hypertension, and Hcy levels. Specifically, we applied a simple mediation model to examine how Hcy, cognitive function, and ALPS are interrelated. Previous research (33) has established an association between Hcy and cognitive function, as measured by MoCA and MMSE scores, with ALPS potentially acting as a mediator in this relationship.

To test this hypothesis, we constructed a mediation model with Hcy as the independent variable (X), cognitive test scores as the dependent variable (Y), and ALPS as the mediator (M). The analysis was conducted using Hayes’ PROCESS macro (model 4) for SPSS (34), controlling for age, gender, and education as covariates.

Notably, given prior evidence that HHT primarily affects executive function and memory, we extracted the corresponding recall subsets from the MMSE and MoCA, averaged them to form a composite recall score, and incorporated this metric into the regression model to specifically analyze these cognitive domains.

## Results

### Demographic and clinical data

Four HHT patients and one normotensive healthy control were excluded due to significant imaging artifacts or unexplained abnormalities in image processing. As shown in Table 1, patients with HHT had significantly higher Hcy load than the controls. The patients also showed significant lower MoCA and MMSE scores. The two groups were comparable on other factors including age, gender, and education (*p* > 0.05).

### Group comparison on ALPS

**Figure 1:**
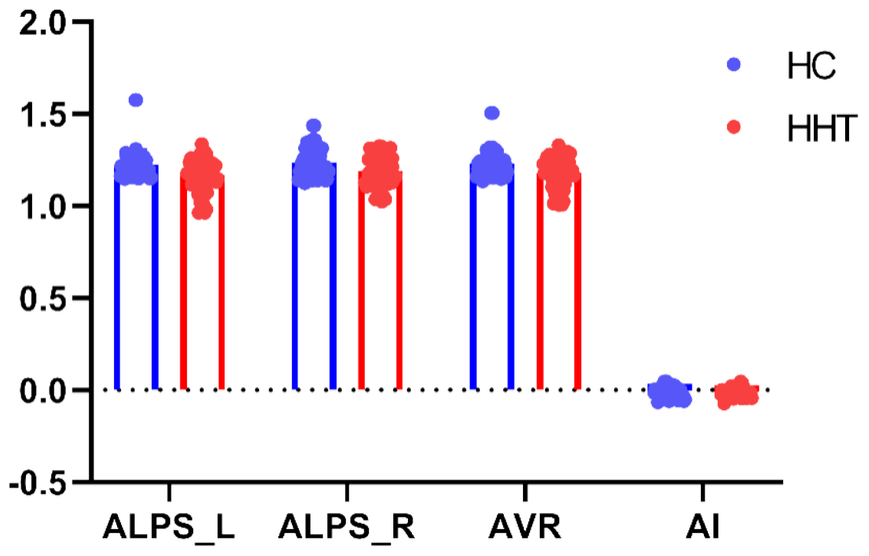
Group comparison on the DTI-ALPS. Scatter plots show group differences on DTI-ALPS between HHT patients and controls. ALPS_L, ALPS_R, AVR, and AI represent the ALPS values of the left hemisphere, the ALPS values of the right hemisphere, the average of both hemispheres, and the asymmetry index (AI, calculated as the difference between the left and right hemispheres divided by the sum of the left and right hemispheres) of the ALPS, respectively.

As shown in Table 2 and Figure 1, compared to normotensive controls, the HHT patients exhibited reduced mean ALPS values in the left hemisphere, right hemisphere, and bilateral hemispheric average, while no statistically significant differences were observed in the hemispheric asymmetry index (AI).

**Table 2:** Group comparison on the diffusion metrics.

Measures	HHT (*n* = 58)	HC (*n* = 50)	*t*	*p*
ALPS_L	1.166 ± 0.086	1.225 ± 0.086	−3.578	0.001^***^
ALPS_R	1.189 ± 0.083	1.235 ± 0.082	−2.904	0.005^*^
ALPS_AVR	1.177 ± 0.080	1.230 ± 0.076	−3.523	0.001^***^
ALPS_AI	−0.010 ± 0.024	−0.004 ± 0.028	−1.165	0.247
D*_xx_* _L_Asso (×10^−3^)	0.7 ± 0.07	0.6 ± 0.05	1.611	0.110
D*_xx_* _L_Proj (×10^−3^)	0.6 ± 0.02	0.5 ± 0.04	2.030	0.047*
D*_xx_* _R_Asso (×10^−3^)	0.7 ± 0.07	0.7 ± 0.04	1.977	0.051
D*_xx_* _R_Proj (×10^−3^)	0.6 ± 0.14	0.6 ± 0.03	2.202	0.031*
D*_yy_* _L_Asso (×10^−3^)	0.7 ± 0.08	0.7 ± 0.05	1.987	0.050
D*_yy_* _L_Proj (×10^−3^)	0.6 ± 0.18	0.5 ± 0.04	2.728	0.008^**^
D*_yy_* _R_Asso (×10^−3^)	0.7 ± 0.08	0.7 ± 0.06	2.648	0.009^**^
D*_yy_* _R_Proj (×10^−3^)	0.6 ± 0.14	0.5 ± 0.04	2.691	0.009^***^
D*_zz_* _L_Asso (×10^−3^)	0.5 ± 0.09	0.4 ± 0.03	3.692	<0.001^***^
D*_zz_* _L_Proj (×10^−3^)	0.8 ± 0.18	0.8 ± 0.04	2.039	0.046*
D*_zz_* _R_Asso (×10^−3^)	0.5 ± 0.08	0.5 ± 0.05	3.450	0.001***
D*_zz_* _R_Proj (×10^−3^)	0.8 ± 0.15	0.7 ± 0.04	2.204	0.031*

ALPS_L, ALPS_R, AVR, and AI represent the ALPS values of the left hemisphere, the ALPS values of the right hemisphere, the average of both hemispheres, and the asymmetry index (AI, calculated as the difference between the left and right hemispheres divided by the sum of the left and right hemispheres) of the ALPS, respectively. *p < 0.05; **p < 0.01; ***p < 0.001.

### Correlation analyses

**Figure 2:**
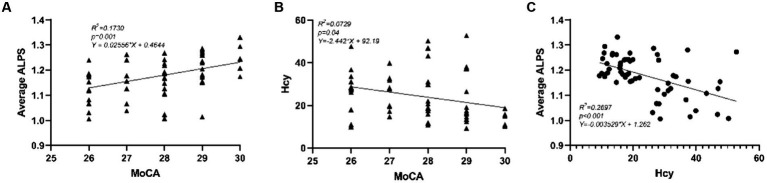
Correlations between **(A)** average ALPS and MoCA, **(B)** between Hcy and MoCA, and **(C)** between average ALPS and Hcy. The scatter plots represent the pairwise correlation analysis between the three variables.

As shown in Figure 2, we performed pairwise correlation analysis of the ALPS index, Hcy levels, and MoCA scores in HHT patients. Significant correlations were found among the three factors. Specifically, higher average ALPS was associated with higher MoCA scores (Figure 2A; *R^2^* = 0.173, *p* = 0.001), while elevated Hcy levels were associated with both lower MoCA scores (Figure 2B; *R^2^* = 0.073, *p* = 0.040) and lower average ALPS (Figure 2C; *R^2^* = 0.270, *p* < 0.001).

### Mediation analysis

**Figure 3:**
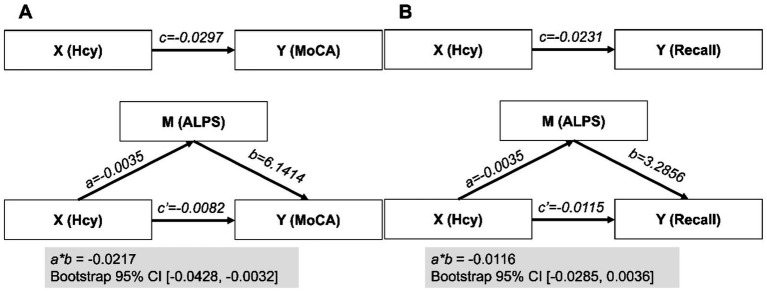
Mediation analyses. In the mediation analyses, the parameter estimates are presented under two scenarios for the dependent variable Y: **(A)** MoCA and **(B)** composite recall. X, M, and Y represent the independent variable, mediator, and dependent variable, respectively; CI, confidence interval.

In a simple mediation model with Hcy as X, ALPS as M, and MoCA as Y, we identified that paths *a* and *c’* were significant, while path *b* was not, suggesting full mediation. The indirect effect accounted for 72.57% of the total effect, meaning the mediation effect represented 72.57% of the total variance. Further bootstrap analysis indicated a 95% confidence interval (−0.0430, −0.0033) that does not include zero, suggesting the mediation effect is statistically significant (Figure 3A). Similarly, when we used the composite recall score as the dependent variable Y, this mediation analysis did not reach statistical significance, as the bootstrap 95% confidence interval included zero, indicating that the mediation effect was not statistically significant (Figure 3B).

## Discussion

This study investigates the relationship between HHT, Hcy levels, and cognitive decline, focusing on the potential role of glymphatic dysfunction. We found that HHT patients exhibited significant reductions in ALPS, which correlated with Hcy levels in a dose-dependent manner. Our results suggest that ALPS, rather than hypertension alone, plays a central role in mediating the relationship between Hcy and cognitive performance. This finding emphasizes the impact of both elevated Hcy and hypertension on glymphatic dysfunction.

Consistent with our hypothesis, we observed a marked decrease in ALPS in HHT patients. This reduction was closely linked to elevated Hcy levels, aligning with findings from previous studies on other neurological conditions such as Alzheimer’s disease, stroke, and sleep disorders, where diminished ALPS has been associated with cognitive decline (30, 31, 35, 36). Despite recent updates to the interpretation of ALPS, which caution against directly equating it with glymphatic function (25), we propose that the observed reduction in the ALPS index among patients, even if not directly indicative of impaired glymphatic function, may at least reflect altered Brownian motion of water molecules along the radial direction surrounding the body of the lateral ventricles.

Additionally, our study highlights the dual impact of hypertension and elevated Hcy in HHT patients, underscoring the potential role of Hcy in influencing glymphatic function. To explore this relationship further, we conducted a mediation analysis, which revealed that Hcy contributes to the observed reduction in ALPS, supporting the hypothesis that Hcy may play a key role in glymphatic dysfunction in this population.

Hypertension and high Hcy are both well-established risk factors for cognitive decline (37). Hypertension contributes to cognitive impairment through mechanisms such as impaired cerebral blood flow, blood–brain barrier disruption, neuroinflammation, and amyloid-beta deposition, for a review see (38). Elevated Hcy further exacerbates these effects by increasing oxidative stress, damaging vascular endothelium, and reducing elasticity, which in turn heightens susceptibility to hypertension. The combination of these factors accelerates the pathogenesis of vascular dementia, Alzheimer’s disease, and other neurodegenerative conditions (39, 40).

Furthermore, elevated Hcy levels have been linked to neurodegeneration through processes like apoptosis, DNA damage, and impaired nerve conduction, all of which contribute to cognitive decline (41). Research has shown that individuals with both hypertension and high Hcy levels exhibit increased white matter hyperintensities (7) and gray matter loss (16), which are indicative of compromised cerebral vasculature and can lead to deficits in spatial learning and synaptic plasticity.

The glymphatic system, which facilitates fluid exchange between cerebrospinal fluid and interstitial fluid, plays a critical role in brain homeostasis. Dysfunction within this system is hypothesized to be a key underlying contributor to VCI (24). Our study adds to the growing body of evidence suggesting that HHT may disrupt lymphatic fluid drainage, potentially exacerbating cognitive decline.

However, the relationship between HHT and glymphatic fluid drainage remains unclear, as does the full extent of HHT’s impact on the brain. Previous studies have shown that hypertension negatively affects brain function, altering brain networks and reducing white matter integrity (18, 42, 43). These findings further highlight the need for protective strategies for individuals at risk of hypertension-induced cognitive decline.

This study has several limitations. First, we used ALPS as a surrogate marker for glymphatic function. While widely used in research, this method has its limitations (25, 44). As noted by the original authors and a recent editorial (25, 44), although ALPS has been widely applied to various brain diseases and is sometimes loosely equated with glymphatic function, its core essence likely reflects the prominent Brownian motion of water molecules in the radial direction at the level of the body of the lateral ventricles. It does not fully capture the entirety of glymphatic function. Moreover, since ALPS primarily measures diffusion in deep white matter, where vasculature and perivascular spaces are minimal, it likely reflects axonal rather than perivascular diffusion. Second, due to technical limitations, the volume of white matter hyperintensities was not included in the analysis in this study. Future research should take into account the contribution of white matter hyperintensities to the differences between ALPS groups. Third, while we assessed global cognition using the MoCA and MMSE, more specialized cognitive domains, such as executive function and processing speed, were not fully explored. While the recall subscores derived from the MMSE and MoCA demonstrated significant associations with Hcy and ALPS, their effects in the mediation model were non-significant. This discrepancy may be attributed to the limited sensitivity of the MMSE recall subtest (due to its simplified scoring) and the restricted variability inherent in the MoCA recall scores. Last but not least, the sample size in this study is relatively small, and larger cohorts will be needed in future research to validate these findings and further investigate the role of glymphatic dysfunction in cognitive decline.

## Conclusion

Our findings reveal that there is a considerable decrease in ALPS in HHT patients, and that this decrease has a dose-effect connection with Hcy levels. Furthermore, we discovered that ALPS, rather than hypertension, largely modulates the link between Hcy and global cognition. These data highlight the importance of both hypertension and elevated Hcy in glymphatic dysfunction.

## Data Availability

The raw data supporting this study will be made available upon request to the corresponding author.

## Footnotes

^1^ www.fmrib.ox.ac.uk/fsl
